# Formation and Fixation of the Annulus of Zinn and Relation With Extraocular Muscles: A Plastinated Histologic Study and Its Clinical Significance

**DOI:** 10.1167/iovs.63.12.16

**Published:** 2022-11-10

**Authors:** Chunjing Ma, Xingyu Zhu, Xuan Chu, Liu Xu, Wei Zhang, Shengchun Xu, Liang Liang

**Affiliations:** 1 Department of Anatomy, School of Basic Medical Sciences, Anhui Medical University, Hefei, China; 2 Hefei Cancer Hospital, Chinese Academy of Sciences, Hefei, China; 3 Human Brain Tissue Resource Center, Anhui Medical University, Hefei, China

**Keywords:** annulus of Zinn, common tendinous ring, extraocular muscles, optic nerve decompression, ultrathin plastination

## Abstract

**Purpose:**

This study aimed to clarify the formation and fixation of the annulus of Zinn (AZ) and its relationship with the extraocular muscles by using ultrathin plastination and three-dimensional models.

**Methods:**

Eighteen cadaveric heads (36 sides of the orbital apex) were plastinated to coronal (16 sides), sagittal (13 sides), and horizontal (5 sides) ultrathin plastination sections to be investigated at both macroscopic and microscopic levels. One cadaveric head was used for endoscopic dissection to identify anatomic landmarks.

**Results:**

There were two fibrous triangles adhered to both ends of the anterior surface of the optic strut. The superior rectus muscle originated from the superior fibrous triangle, and the lateral, inferior, and medial rectus muscles emerged from the inferior fibrous triangle. It was not until 5.46 ± 0.41 mm anterior to the optic strut that the complete tendinous ring composed of rectus muscles, optic nerve sheath, and periosteum was formed. The superior oblique and levator palpebrae superioris muscles originated from the medial fibrous band of the AZ. At the posterior of the AZ, there was a potential passage between the medial rectus muscle and the optic nerve.

**Conclusions:**

The fixation of the AZ was composed of the connection of the annular tendon to the optic strut posteriorly and the attachment of the complete tendinous ring to the lesser and greater wings of the sphenoid bone anteriorly. The triangular route area between the optic nerve and medial rectus muscle was located on the anterior side of the base of the optic strut.

The annulus of Zinn (AZ), also known as the common tendinous ring,^1^ is a fibrous ring that surrounds the optic canal and part of the superior orbital fissure at the orbital apex and gives origin to the four rectus muscles.

Some lesions occurring in the orbit, such as traumatic optic neuropathy^2^ and thyroid dysfunction optic neuropathy,^3^ may lead to compression and edema of the optic nerve (ON) in the orbital apex. In these cases, the expansion space of the ON may be restricted by the anatomic structure of the AZ.^2^^,^^4^ Endoscopic transnasal ON decompression is usually required as an adjuvant therapy method when drug therapy fails. However, there is no consensus on whether to incise the AZ in ON decompression surgery.^3^^,^^5^^–^^7^ It has been suggested^2^^,^^4^^,^^7^ that, in addition to the incision of the optic nerve sheath, the AZ should be incised to completely decompress the ON, whereas some authors^8^^,^^9^ have proposed that the integrity of the AZ should be maintained and avoid damage to it during the operation. As the origin of extraocular muscles, the AZ plays an important role in surgical planning in the orbital apex. Nevertheless, the definition of AZ and its relationship to extraocular muscles is still ambiguous in the anatomic and clinical literature.^10^^–^^15^ Clarifying this issue may facilitate analyzing the impact of incising the AZ on the rectus muscles to choose a safer surgical approach. In addition, the ON and medial rectus muscle (MR) are the critical structural and anatomic landmarks when decompressing near the AZ of the orbital apex during endoscopic surgery.^16^ It is vital for accessing the medial space in endoscopic surgery to study the relationship between the ON and MR in the area of the AZ.

Therefore, the formation and fixation of the AZ and its relationship with extraocular muscles were analyzed, and the relationship between the ON and MR in the region of the AZ was also investigated in this study by using a combination of ultrathin plastination and three-dimensional models.

## Methods

A total of 18 cadaveric heads (16 males and 2 females; age range, 35–76 years; average age at death, 63 years) were used in this study. The cadavers were donated to the Department of Anatomy (Anhui Medical University, China) for education and research. This study was approved by the University Ethics Committee.

### Ultrathin Plastination

Tissue blocks containing orbital apex were collected. These blocks were dehydrated in acetone at –25°C for 4 weeks and then degreased at room temperature for 6 weeks. Afterward, the tissue blocks were impregnated with a resin mixture of E12/E6/E600 (Biodur, Heidelburg, Germany). After curing in an oven at 60°C for 7 to 10 days, the cured tissue blocks were cut in the coronal (16 sides), sagittal (13 sides), and horizontal (5 sides) planes with the Exakt 310 CP cutting system (Exakt, Norderstedt, Germany). The average thickness of the sections was approximately 200 to 250 μm. These sections were stained with Stevenel's blue and Alizarin red S. The stained sections were scanned with an Olympus BX 63 microscope (Olympus, Tokyo, Japan).

### Three-Dimensional Reconstructions and Endoscopic Dissections

The sagittal and horizontal sections were imported into 3D Slicer software (a free, open-source platform; http://www.slicer.org) to reconstruct after calibrating and uniforming resolution. Different colors were chosen to colorize various parts of the sections to define distinct structures, such as bones, nerves, blood vessels, and fibrous tissue, during image processing.

One cadaveric head (two sides) was prepared for anatomic dissection. An expended endoscopic endonasal approach was performed using 30° endoscopes (SHREK, Shanghai, China) connected to a digital video recorder. After bilateral middle turbinectomy, nasal septectomy, and ethmoidectomy, the sphenoid sinus was opened to expose the orbital apex region. The anatomic landmarks of the orbital apex were identified and recorded using digital video.

### Measurements

The length from the orbital opening of the optic canal to the common tendinous ring was selected for measurement and analysis to investigate the relationship between the ON and MR. The horizontal distance from the complete fibrous ring formed by rectus muscles to the anterior border of the optic strut was measured. The vertical distance of the ON and MR and its projection distance on the corresponding medial orbital wall (window distance) at the anterior border of the optic strut were also measured. The area of the triangle formed by the ON, MR, and the optic strut (vertical triangle), as well as its projection area on the corresponding bony medial orbital wall (window triangle), was calculated, respectively. Additionally, the vertical distance from the ophthalmic artery to the window triangle at the anterior border of the optic strut was also measured. The measurements were directly conducted in coronal sections, while sagittal and horizontal section measurements were taken using three-dimension slicer software.

## Results

### Origin of the Extraocular Muscles and Formation and Fixation of the Common Tendinous Ring

**Figure 1.**
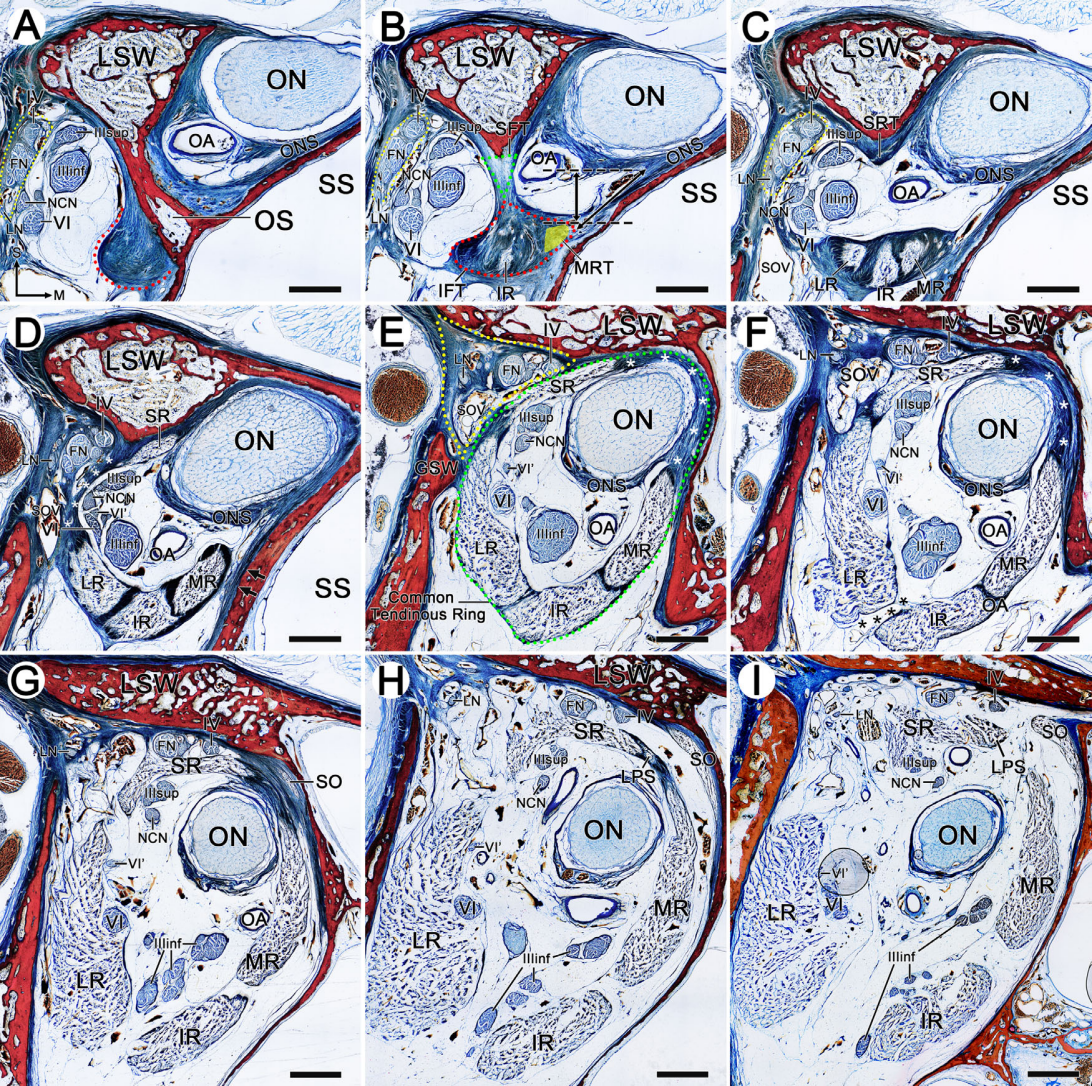
Origin of rectus muscles, LPS and SO, and the formation of the common tendinous ring in the orbital apex. Plastinated sections from the cadaveric head of a 67-year-old male. (**A**) The coronal section passing through the OS. (**B–I**) The coronal sections of 1 mm, 2 mm, 4 mm, 6 mm, 7 mm, 9 mm, 11 mm, and 14 mm anterior to the OS, respectively. (**A**) An annular tendon (*red semicircle dotted line*) was attached to the inferolateral side of the OS. The ON and OA were located on the superomedial side of the OS, and cranial nerves (CNs) IV, VI, III, FN, LN, and NCN were contained in the lateral space of the OS. Laterally, CNs IV, FN, and LN were arranged into a long strip shape (*yellow ellipse dotted line* in **A–C**). (**B**) The SFT (*green dotted line*) was connected to the IFT (*red dotted line*). The SFT was attached to the anterior surface of the upper OS, while the IFT was mostly attached to the inferolateral surface of the lower OS. The fibers of the IR could be seen. The position of the MR (*yellow triangle area*) was inferred based on the section of 2 mm anterior to the OS because the muscle bundles of the MR have not yet appeared. Two *black dotted lines* were drawn at the lowest point of the ONS and the vertex of the MR, respectively, and the vertical distance was measured. The window distance (*oblique double-headed arrow*) between the ON and MR was also measured. (**C**) The SFT of the SR separated from the IFT and attached to the inferior margin of the LSW, and the muscle bundles of the MR, IR, and LR emerged from the IFT. Medially, the SRT fused with the superolateral side of the ONS. (**D**) Laterally, the LR fused with the lateral tendon of the SR to form a lateral fibrous band (*white asterisk*). Medially, the tendon of the MR did not tightly attach to the lateral wall of the sphenoid sinus (*arrows*). Note that the CN VI gave rise to an early branching (VI′). The CNs III, VI, VI ′, and NCN entered intraorbital space, while CNs IV, FN, and LN entered extraorbital space. (**E**) The MR fused with the inferomedial part of the ONS to form the medial fibrous band (*white asterisk*). The common tendinous ring (*green ellipse dotted line*) was formed by four rectus muscles, periosteum, and ONS. Laterally, the CNs IV and FN coursed along the superolateral aspect of the SR in triangular extraorbital space (*yellow dotted line*). The SOV was tightly attached to the lateral fibrous band. (**F**) The tendon connection between the LR and IR was separated (*black asterisk*). The medial fibrous bands between the SR and MR thickened (*white asterisk*). (**G**) The SO was tightly attached to the superomedial aspect of the orbital wall. (**H**) The LPS emerged from the medial fibrous band between the SR and MR. (**I**) More anteriorly, the superomedial side of the common tendinous ring separated, and the muscle bundles of the LPS coursed along the superomedial aspect of the SR. In addition, before the formation of the complete fibrous ring, there was a potential space between the MR and ON in the lateral wall of the SS, which was filled with loose adipose tissue in **B–E**. FN, frontal nerve; GSW, greater wing of the sphenoid bone; IFT, inferior fibrous triangle; IIIinf, inferior division of the oculomotor nerve; IIIsup, superior division of the oculomotor nerve; IV, trochlear nerve; LN, lacrimal nerve; LSW, lesser wing of the sphenoid bone; M, medial; MRT, tendon of the medial rectus muscle; NCN, nasociliary nerve; OA, ophthalmic artery; ONS, optic nerve sheath; OS, optic strut; S, superior; SFT, superior fibrous triangle; SOV, superior ophthalmic vein; SRT, tendon of the superior rectus muscle; SS, sphenoid sinus; VI, abducens nerve; VI′, early branching of the abducens nerve. *Bar:* 2 mm.

**Figure 2.**
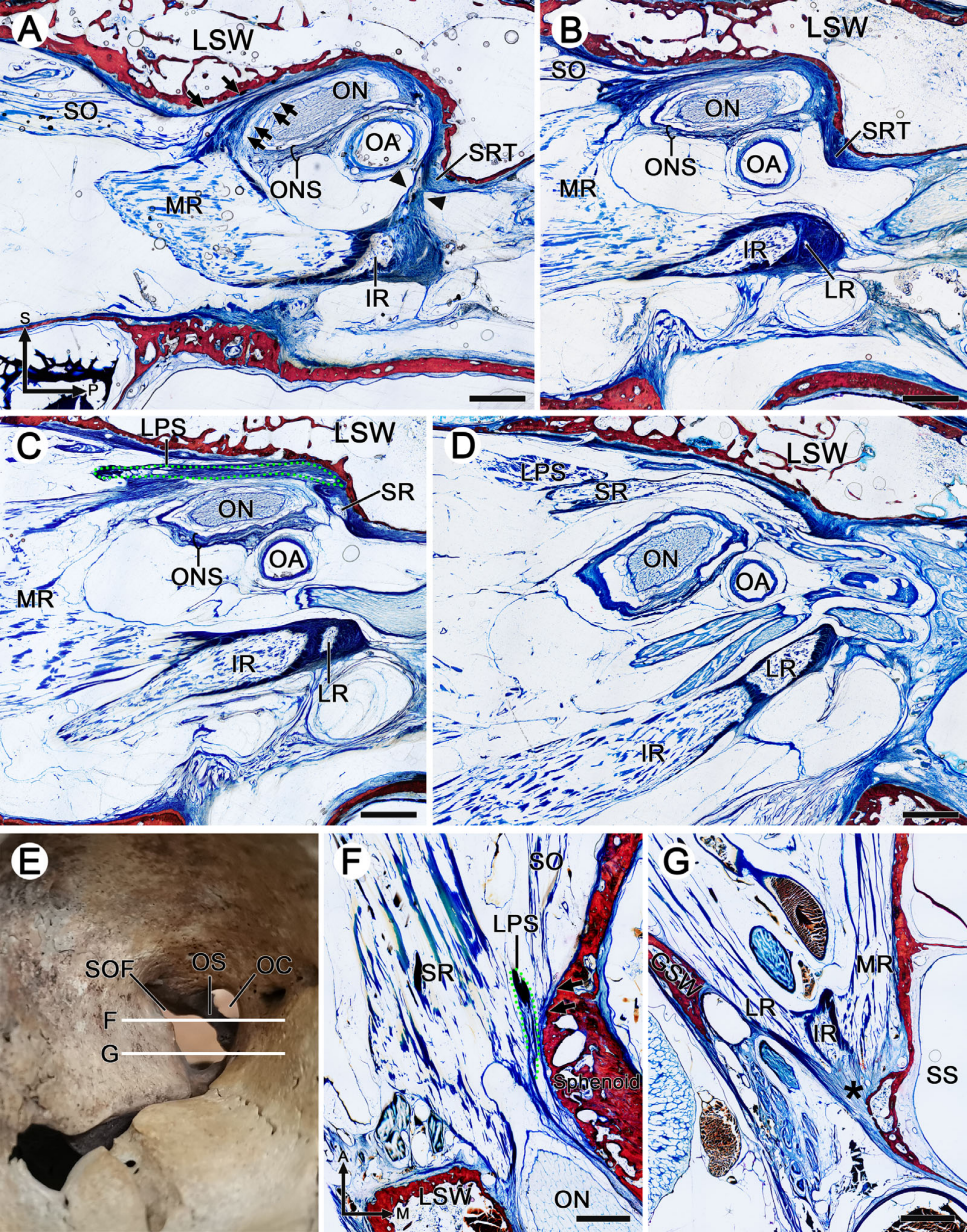
Plastinated sections from cadaveric heads of a 77-year-old male (**A–D**) and a 75-year-old male (**F–G**). (**A–D**) Sagittal sections of 1 mm, 2 mm, 3 mm, and 5 mm of the lateral side of the OS connected with the body of the sphenoid bone. (**F**, **G**) Corresponding to the horizontal sections in **E**. (**A**) Posteriorly, the SRT was connected to the tendon of the MR, IR, and LR (*arrowheads*). Anteriorly, the muscle bundles of the MR widened superiorly to fuse with the ONS. The tendon of the SO was embedded superiorly to the tendon complex (*double arrows*) formed by the ONS and MR and attached to the inferior margin of the LSW (*arrows*). (**B**) The connection between the SRT and the tendon of the MR, IR, and LR separated, and the muscle bundles of the MR enlarged and widened anteriorly. (**C**) The muscle bundles of the SR were visible, and the tendon of the LPS (*green dotted line*) fused with the tendon of the SR and MR but attached to the anterosuperior aspect of the SR. (**D**) Laterally, the LPS moved anteriorly along with the SR. (**F**) The tendon of the LPS (*green dotted line*) was visible and fused with the ONS and medial side of the SR. The attachment point of the SO was located on the orbital wall (*arrows*) in the anteromedial aspect of the SR and LPS. (**G**) The tendon of MR, IR, and LR fused posteriorly (*black asterisk*). A, anterior; OC, optic canal; P, posterior. *Bar:* 2 mm.

**Figure 3.**
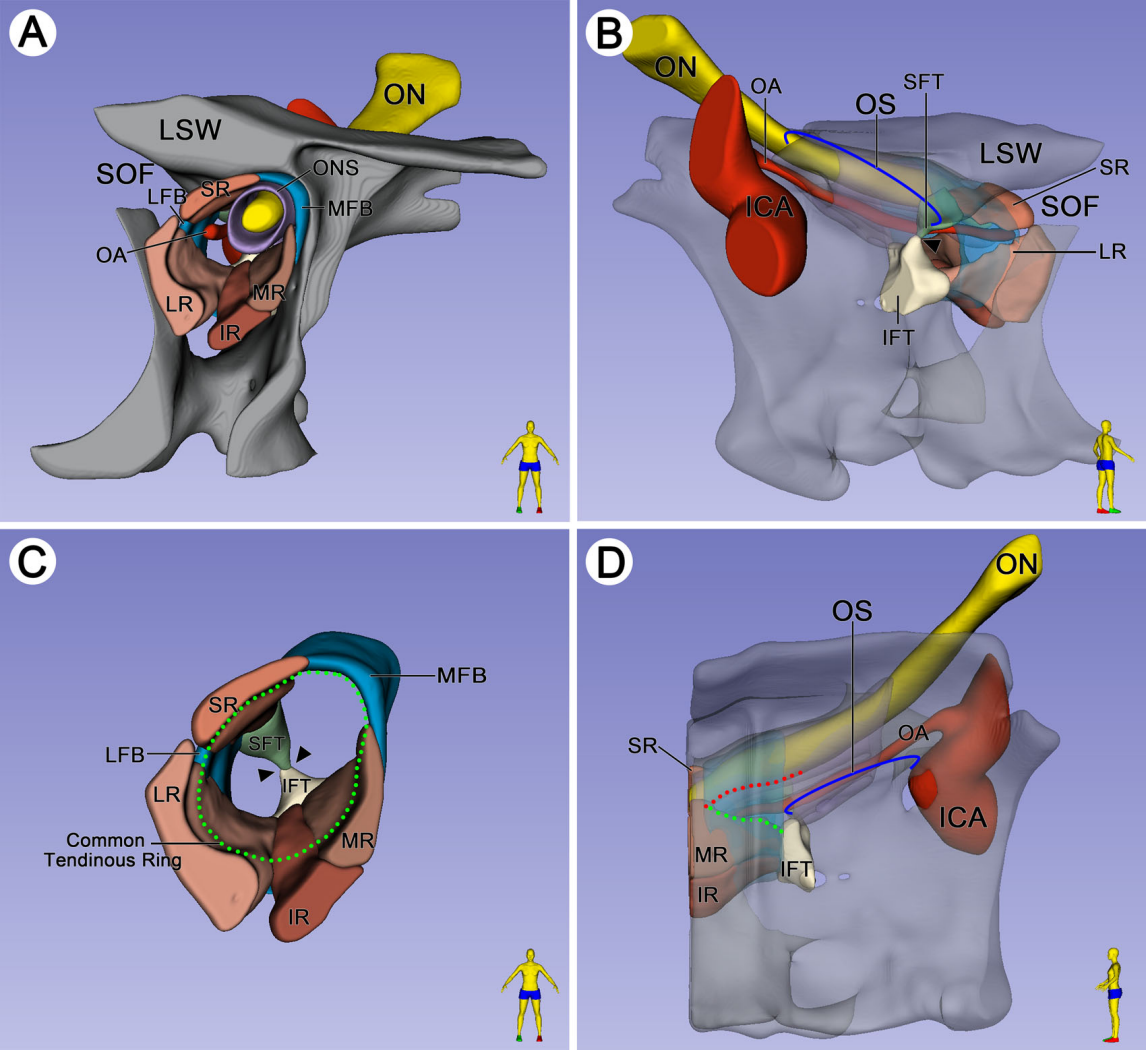
Plastinated sections used for three-dimensional reconstruction were obtained from the cadaveric head of a 76-year-old male. (**A**) Spatial relationship of the vital structures of the orbital apex on the three-dimensional reconstructed model. (**B**) The SFT was located inferior to the LSW, while the IFT was attached to the inferolateral of the OS. As the SR extended posteriorly, the SFT of the SR fused with the IFT of the LR, IR, and MR (*arrowheads*). (**C**) With the muscle bundles of the SR, LR, IR, and MR extended anteriorly, the common tendinous ring (*green dot line*) formed based on rectus muscles with the help of the LFB and MFB. (**D**) Medially, there was a potential passage between the inferior border of the ON (*red dotted line*) and the superior border of the MR (*green dotted line*) anterior to the OS. ICA, internal carotid artery; LFB, lateral fibrous bands; MFB, medial fibrous bands; SOF, superior orbital fissure.

The formation of the common tendinous ring and the origin from the extraocular muscles were observed on coronal, sagittal, and horizontal serial sections (Figs. 1 and 2; cadaveric orbit from 67-, 77-, and 75-year-old males, respectively). The inferolateral aspect of the optic foramen was bounded by the optic strut (the posterior root of the lesser wing of the sphenoid bone). The dumbbell-shaped optic strut separated the optic canal (with superomedial position to the strut) from the superior orbital fissure (with inferolateral location to the strut). An annular tendon was tightly attached to the periosteum of the inferolateral surface of the sphenoidal part of the optic strut and the surface of the sphenoid body (Figs. 1A, 2G). On the anterior surface of the optic strut, there were two fibrous triangles adhered to both ends of this dumbbell-shaped structure. The tendon of the superior rectus muscle (SR) originated from the superior fibrous triangle, and tendons of the MR, lateral rectus muscle (LR), and inferior rectus muscle (IR) emerged from the inferior fibrous triangle (Figs. 1B, 2A, 3B, 3C). These rectus muscle bundles extended anteriorly and gradually became thicker and wider. The superior fibrous triangle surrounded the SR to form a single superior fibrous ring and fused with the superolateral part of the optic nerve sheath, while the inferior fibrous triangle surrounded the LR, IR, and MR to form a three-compartment inferior fibrous ring complex (Fig. 1C). Laterally, the LR extended superolaterally, and at a distance of 4 mm anterior to the optic strut, the fibrous rings of the LR and SR fused with each other to form a lateral fibrous band connection, which divided the orbit into a triangular extraorbital space and an oval intraorbital space (Figs. 1D, 1E). Medially, however, it was not until 5 to 6 mm anterior to the optic strut that the widened MR began to fuse with the inferomedial part of the optic nerve sheath (Figs. 1E, 2A), and as a result, the common tendinous ring (complete fibrous ring), composed of the fibrous rings of individual rectus muscles, optic nerve sheath, and periosteum, was formed in the orbital apex (Figs. 1E, 3C).

This complete fibrous ring was stuck laterally to the greater wing of the sphenoid bone by the LR, adhered superiorly to the lesser wing of the sphenoid bone by the SR and the optic nerve sheath, and attached medially to the body of the sphenoid bone by the optic nerve sheath and the MR, whereas its inferior aspect floated on the orbital fat (Fig. 1E). Nevertheless, this complete fibrous ring extended only 1 to 2 mm anteriorly, and then an adipose channel first appeared between the LR and the IR due to the separation of them (Fig. 1F). As the ON moved toward the center of the orbit, the optic nerve sheath gradually separated from the common tendinous ring, and then the SR and the MR connected together with a medial fibrous band, from which the levator palpebrae superioris muscle (LPS) originated. At the same time, the common tendinous ring began to separate from the orbital wall. The muscle bundles of the superior oblique muscle (SO) emerged from the periosteum of the superomedial side of the medial fibrous band between the SR and MR (Figs. 1G, 1H). Anteriorly, following the separation of the medial fibrous band between the SR and MR, the muscle bundles of the LPS moved superolaterally (Fig. 1I). As the MR gradually widened superiorly to fuse with the optic nerve sheath, the SO tendon was inserted into the superior aspect of both (Figs. 2A, 2B), while the LPS tendon appeared on the lateral side of the SO and also fused with the superior aspect of the MR (Figs. 2C, 2F). The LPS tendon was attached to the anterosuperior side of the SR and moved along the superior aspect of the SR (Figs. 2C, 2D).

### Cranial Nerves and Vasculature of the Orbital Apex

On the anterolateral side of the orbital opening of the optic canal, the trochlear, frontal, and lacrimal nerves were arranged in a long strip shape based on the order of the superior to inferior. The two branches of the oculomotor nerve were located medial to the long strip-shaped nerve group, while the nasociliary and abducens nerve were located on the inferolateral side of the oculomotor nerve. These nerves were crowded together in the superior orbital fissure (Figs. 1A–1C). As the lateral fibrous band formed between the LR and SR, the trochlear, frontal, and lacrimal nerves were divided into the triangular extraorbital space. The superior ophthalmic vein was tightly fixed between the periosteum and the lateral fibrous band. The oculomotor, nasociliary, and abducens nerves entered the intraorbital space (Figs. 1D, 1E). More anteriorly, the trochlear and frontal nerves coursed together along the superior orbital wall, while the lacrimal nerve ran along the superolateral side in extraorbital space. The superior branch of the oculomotor nerve coursed toward the SR, while its inferior branch was once again divided into three branches that traveled inferiorly and medially. The nasociliary nerve, which was located between the abducens and oculomotor nerves, ran along the lateral side of the ON and went to the superior aspect of the ON (Figs. 1F–1I). In the superomedial aspect of the optic strut, the ophthalmic artery was located in the inferolateral aspect of the ON, and it coursed inferolaterally after leaving the optic canal. After the ophthalmic artery traveled a certain distance, the ophthalmic artery gradually turned inferomedial of the ON when the MR fused with the optic nerve sheath (Figs. 1A–1E).

### Relationship Between the ON and MR

**Figure 4.**
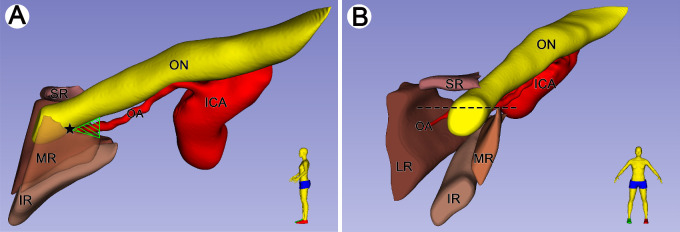
Spatial relationship between ON and MR on the three-dimensional reconstructed model. Plastinated sections used for three-dimensional reconstruction were obtained from the cadaveric head of a 39-year-old male. (**A**) The ON coursed inferolaterally along with the ophthalmic artery. Posteriorly to the pentagram, the ON was located superiorly to the MR. There was a potential space between the ON and MR (*green triangle area*). (**B**) Anterior to the position of the pentagram in **A**, the ON intersected the MR (*black dotted line*), locating the superior portion of the MR.

**Figure 5.**
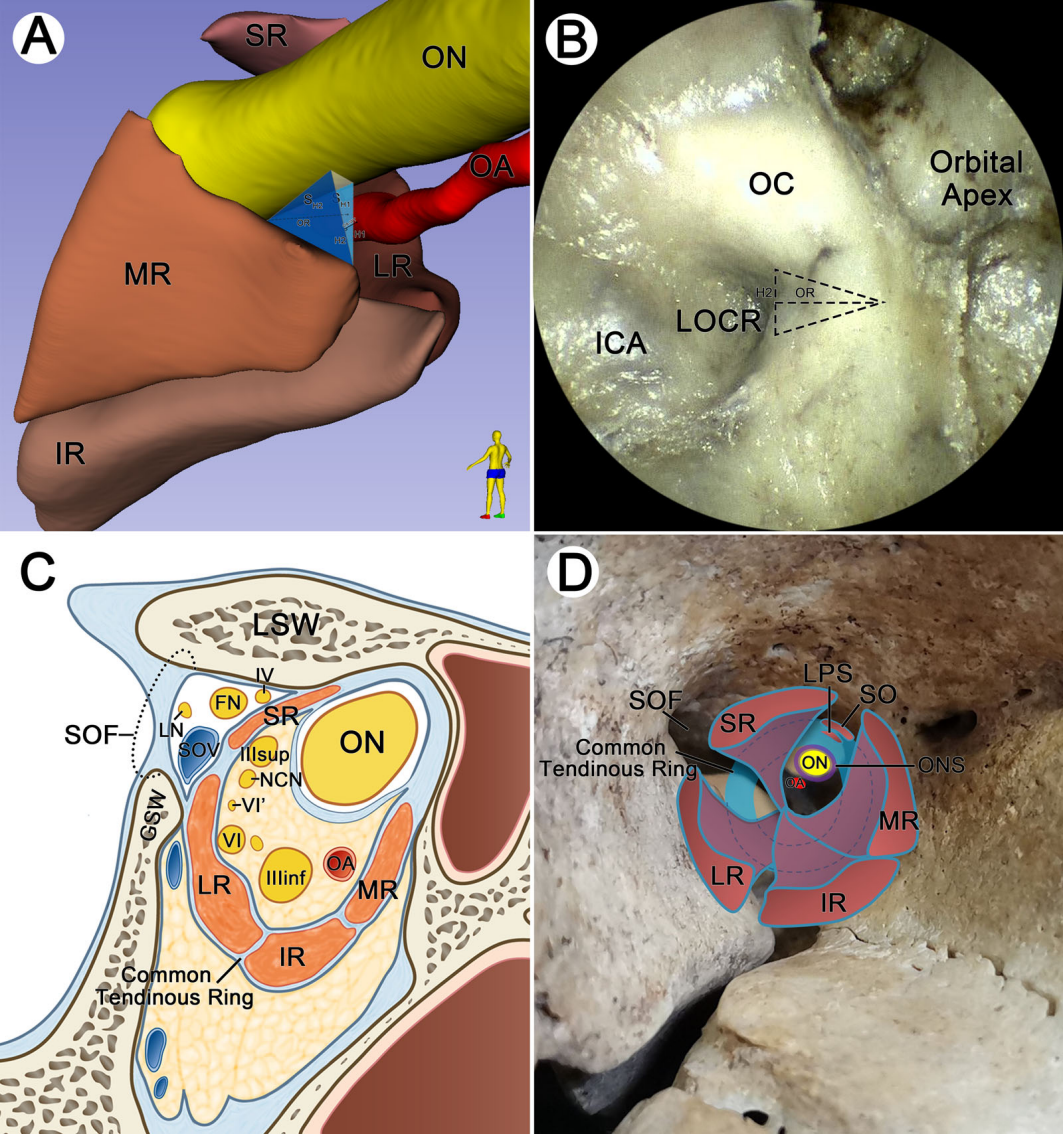
Three-dimensional model in **A** from the same cadaveric head in Figure 4. Specimen in **B** from the cadaveric head of a 75-year-old male. (**A**) Diagram of measurement on the three-dimensional reconstructed model. H1 and H2 represent vertical and window distances, respectively. S_H1_ and S_H2_ are vertical and window triangular regions, respectively. OR represents the horizontal distance from the orbital opening of the optic strut to the formation of the common tendinous ring. OA-WT is the vertical distance of the OA to the window triangle. (**B**) The anatomic landmarks in the region of the orbital apex under endoscopic view. The anterior triangle of the optic strut was speculated (*triangle dotted area*). (**C**) The schematic diagram of Figure 1E shows the relationship between the common tendinous ring and vital cranial nerves. (**D**) Schematic diagram of the relationship between the rectus muscles, LPS, SO, and the common tendinous ring. LOCR, lateral optic carotid recess.

The plastinated sections from two different cadaveric heads (76- and 39-year-old males, respectively) were used for three-dimensional reconstruction in Figure 3 and Figure 4. After the ON left the optic canal, the ON coursed inferolaterally and entered the intraorbital space. The MR emerged from the inferior fibrous triangle and gradually widened superiorly until it fused with the inferomedial aspect of the ON. Anteriorly to the optic strut, there was a potential space of loose connective tissue between the ON and MR before the MR fused with the optic nerve sheath (Figs. 1B–1E). In three-dimensional models, the triangular passage represented the potential space (Figs. 3D, 4A), which was defined as the anterior triangle of the optic strut in this study. The muscle bundles of the MR stretched and widened superiorly to encircle the ON anterior to the passage (Fig. 4). According to the results of endoscopic dissections (cadaveric head from a 75-year-old male), the optic canal, internal carotid artery, lateral optic carotid recess, and orbital apex were recognized, and the location of the anterior triangle of the optic strut was also speculated (Fig. 5B).

The measurement data were obtained from plastinated sections of 17 cadaveric heads (34 sides). The mean distance from the ring structure to the optic strut was 5.46 ± 0.41 mm. The mean vertical and window distance between the ON and MR anterior to the optic strut was 1.97 ± 0.60 mm and 3.29 ± 0.86 mm, respectively. The mean area of the vertical triangle and window triangle was 5.39 ± 1.76 mm^2^ and 9.01 ± 2.53 mm^2^, respectively. The mean distance from the ophthalmic artery to the window triangle was 1.18 ± 0.36 mm (Fig. 5A and Table).

**Table.** Measurement in Relation to the ON and MR

Measurement	Range (*n* = 34)	Length, Mean ± SD (*n* = 34)
OR, mm	5.0–6.0	5.46 ± 0.41
H1 (vertical distance), mm	1.0–3.5	1.97 ± 0.60
H2 (window distance), mm	1.4–4.6	3.29 ± 0.86
S_H1_ (vertical area), mm^2^	2.5–10.5	5.39 ± 1.76
S_H2_ (window area), mm^2^	3.5–13.8	9.01 ± 2.53
OA-WT, mm	0.5–2.3	1.18 ± 0.36

OA-WT, vertical distance of the ophthalmic artery to the window triangle; OR, horizontal distance from the orbital opening of the optic strut to the formation of the common tendinous ring.

## Discussion

### Controversy About the Origin of the Extraocular Muscles and AZ

Currently, the origin of the extraocular muscles and the definition of the AZ are still described inconsistently in the literature.^10^^–^^13^^,^^15^^,^^17^ Burkat and Lemke^17^ reported that the extraocular muscles, except the inferior oblique muscle, were derived from the AZ. According to Matsuo et al.,^10^ the AZ was believed to be the origin of the four rectus muscles. Recently, an independent origin of the SR was first proposed by Naito et al.^11^ in their study. However, evidence of the origin of the extraocular muscles and the structure of the AZ is difficult to exhibit due to the technical limitations of gross anatomy and histology. Therefore, a combination of ultrathin plastination and three-dimensional models was used to accurately display these structures in this study.

The classic description of the AZ^11^^,^^12^^,^^18^ mentions that the annulus mainly includes two parts: (1) the upper portion of the origin of the SR and variable superior head of the LR and (2) the lower part of the origin of the LR, MR, and IR. However, a more detailed description of the AZ was not obtained. Hence, further information about the formation of the AZ was demonstrated in our results. According to our findings, the fixation of the common tendinous ring mainly comprises the connection of the annular tendon to the optic strut posteriorly and the attachment of the complete fibrous ring to the lesser and greater wings of the sphenoid bone anteriorly. The complete fibrous ring (Figs. 1E, 3C, 5C) was formed by the interconnection of the rectus muscles during the process of extending anteriorly, which is located approximately 5.46 ± 0.41 mm anterior to the orbital opening of the optic canal, the length extending approximately 2 mm. Lacey et al.^15^ measured the length of the AZ to be approximately 10 mm in their investigation. The result they measured may be the annular structure observed by surgeons in the actual anatomic operation. Nevertheless, the part of the length they observed anatomically may come from the annular structure produced by the connection of the surrounding periosteum rather than the complete fibrous ring formed by the rectus muscle. The actual length of the AZ is much shorter than the anatomically shown ring structure due to the incomplete fusion of the MR with the inferomedial side of the optic nerve sheath posteriorly. As Kanehira et al.^12^ explained, the AZ was most likely caused by dissection rather than the histologic entity.

The annular tendon located on the inferolateral aspect of the optic strut was described as the origin of all of the IR and part of the LR and MR,^19^ while Rouvière^20^ stated that the tendon was divided into six parts, from which the muscles arise. The superior tendon was described by Lockwood^21^ as giving origin to part of the LR, all of the SR, and part of the MR. However, Naito et al.^11^ suggested that the superior tendon is an independent origin of the SR in their fetus study. In fact, in this study, the origin of all the LR, IR, and MR was derived from the annular tendon, and the superior tendon was only the origin of the SR. Besides, the SR tendon fused with the annular tendon when it extended posteriorly and attached to the inferolateral aspect in the optic strut. Consequently, the rectus muscles should be regarded as having a common origin in this research. Perhaps the independent origin of the SR in fetuses shifts to a common origin in adults.^12^ The muscle bundle of the SR emerged from the superior fibrous triangle and gradually extended anteriorly. The lateral side of the SR was fused with the superior edge of the LR to form a lateral fibrous band, while the medial side of the SR was connected with the MR by optic nerve sheath and periosteum to form a medial fibrous band. Due to the connection between the SR and LR laterally, the LR was thought to have “two heads.”^13^^,^^15^^,^^18^ However, it has been reported^19^ that this description gives exaggerated facts, noting that the superior edge of the LR was fused at the superior orbital fissure with the inferior edge of the SR, and similarly, the inferior edge blended with the IR. This viewpoint was supported by our results. The findings showed that the complete fibrous ring formed by the four rectus muscles was firmly attached to the superomedial side of the orbital wall. The SO and LPS emerged from the medial fibrous band, which was formed by the tendons of the SR and MR, periosteum, and the optic nerve sheath (Fig. 5D). The attachment point of the LPS is thought to be located on the superomedial aspect of the fibrous ring because the muscle bundles of the LPS emerge from between the SR and MR. However, the attachment point of the SO is located superomedial to the orbital wall anterior to the fibrous ring.

The function of the extraocular muscles seems to originate from the pulley system of the eyeball,^22^^–^^24^ although the work from the Demer group^25^ suggested that the direction of movement of the eyeball may also be related to the origin of the extraocular muscles at the posterior orbital apex. Piccirelli et al.^26^ found that the greatest deformation of the extraocular muscles during eye horizontal movement occurred at the orbital apex rather than where the muscle was inserted into the sclera. Exploring the origin and attachment of the extraocular muscles at the orbital apex may aid in better understanding the mechanics of the extraocular muscles and clarifying the etiologies of ocular misalignment.

### Possible Risk of Incising AZ

The AZ restricts the expansion space of the ON during endoscopic transnasal optic canal decompression.^4^ Some literature^2^^,^^4^^,^^7^ has reported that incising the AZ was necessary during decompression. However, the risks and benefits of incising the AZ are still unclear due to limited reports.

Chen et al.,^4^ Lin et al.,^2^ and Mesquita Filho et al.^7^ believed that the AZ should be incised to completely decompress the ON in addition to incising the optic nerve sheath during endoscopic transnasal optic canal decompression. However, Yang et al.^9^ and Di Somma et al.^8^ suggested that the integrity of the AZ should be maintained and avoid damage to it during the operation. Tu et al.,^3^ in their series of cases, incised the AZ for further decompression of the orbital apex. Despite mentioning the occurrence of postoperative diplopia, the authors failed to state what led to it. The damage to the AZ may be one of the reasons. Sowerby et al.^6^ conducted the posterior extent of decompression that reached the AZ in traumatic ON patients, and in their cases, three of the four patients had preoperative diplopia and remained postoperatively. Singh et al.^5^ started the operation anterior to the AZ and extended anteriorly rather than incise the AZ in the decompression of the orbital apex, and there were also no postoperative complications.

In the position of the complete fibrous ring formed by rectus muscles, the ON was being immobilized by the medial fibrous band, and the SO and LPS also emerged from the medial fibrous band. The complete fibrous ring, which is attached anteriorly to the lesser and greater wings of the sphenoid bone, is one of the fixations of the AZ. Two situations may occur if the complete fibrous ring is incised: (1) incision on the medial aspect may directly injure the muscle fibers of the MR, and (2) incising the medial fibrous band may damage the ON, the origin of the SO and LPS, as well as impair the stability of the common tendinous ring. Both conditions may lead to diplopia.^3^^,^^6^ Besides, the optic strut was proposed to be removed for resection of the anterior clinoid process via transcranial or endoscopic approaches.^27^^,^^28^ However, removal of the optic strut may be risky due to the attachment of the AZ to the optic strut. In the orbital opening of the optic canal, the superior and inferior fibrous triangle fused into an annular tendon and were attached to the inferolateral aspect of the optic strut. The optic strut serves as the other attachment point of the fixation of the AZ, and its removal is also likely to damage the stability of the extraocular muscles in the orbital apex and lead to diplopia. In summary, it might be unwise to incise the AZ or remove the optic strut because it might undermine the stability of the common tendinous ring and increase the likelihood of diplopia.

### Spatial Relationship Between the ON and MR

There are many studies on the relationship between ON and MR in the literature,^29^^–^^32^ but research in the area of the AZ is rarely involved. Bleier et al.^29^ defined the superomedial region near the AZ as “Zone C” in their study. They also reported that this area of operation is extremely challenging in technique, noting that the distance between the ON and MR may be less than 1 mm. According to our measurement results (Table), indeed, this space is very limited, and any operation is performed in the region with extreme caution. Petrov et al.^31^ studied the positional relationship between the ON and MR, helping surgeons to identify the ON in advance to prevent damage to it. However, it was difficult to study the relationship between the ON and MR in the position of the AZ due to the limitation of the scanning distance and the complexity of the neurovascular and tissue. Therefore, this research investigated the relationship between the ON and MR in the area of the AZ. A potential passage between the ON and MR was observed in this study. In addition, we also measured the vertical and window area of the anterior triangle of the optic strut in 34 cases (Table), which may provide reliable data for clinical surgery. From an endoscopic perspective, the anterior triangle of the optic strut was located anterior to the orbital opening at the base of the optic strut (Fig. 5B), between the inferior border of the ON and the superior border of the MR. The base of the optic strut is endocranially related to the lateral optic carotid recess.^33^ The lateral optic carotid recess has been reported as an anatomic marker in endoscopic surgery.^34^ The anterior triangle of the optic strut may be employed as an entry point into the optic canal or the orbital apex. The heat damage to the ON^35^ caused by drilling the optic canal may be prevented by drilling the anterior triangle of the optic strut. However, the distance between the ophthalmic artery and the lateral wall of the sphenoid sinus, as well as a lost or fragmented bony septum of the optic canal facing the sphenoid sinus,^36^ should be taken into account. Besides, the abducens nerve is one of the important structures to avoid injury in surgical procedures in the orbital apex. Wysiadecki et al.^37^^,^^38^ described the variation of the intracranial and cavernous segment of the abducens nerve. Although we have not found the duplication or early branching of the abducens nerve in the fissural segment, one case of the early branching of the orbital segment of the abducens nerve was found in this study. A comprehensive awareness of the variations of the abducens nerve may facilitate reducing the risk of complications associated with surgical procedures.

### Study Limitations

The number of specimens used in this study was relatively small, and most of them were all aged specimens. Studies^11^ have shown that the origin of extraocular muscles is different in fetuses and adults. There could be differences in the formation of the AZ as well. Nevertheless, fetal specimens are difficult to obtain for this study. Besides, although the ultrathin plastination exhibited the fine structures of the orbital apex, the process of obtaining these sections was time-consuming.

## Conclusions

The complete fibrous ring was formed by the tendons of the rectus muscles, the optic nerve sheath, and the periosteum. The fixation of the common tendinous ring was composed of the connection of the annular tendon to the optic strut posteriorly and the attachment of the complete fibrous ring to the lesser and greater wings of the sphenoid bone anteriorly. There was an anterior triangle of the optic strut between the ON and MR in the posterior of the complete fibrous ring. This triangular route area was located on the anterior side of the base of the optic strut, which corresponded endocranially to the lateral optic carotid recess.
